# Coherent control of electron-ion entanglement in multiphoton ionization

**DOI:** 10.1038/s41377-025-02151-y

**Published:** 2026-03-06

**Authors:** Yi-Jia Mao, Zhao-Han Zhang, Yang Li, Takeshi Sato, Kenichi L. Ishikawa, Feng He

**Affiliations:** 1 Tsung-Dao Lee Institute, Shanghai Jiao Tong University, Shanghai, China; 2 Key Laboratory for Laser Plasmas (Ministry of Education) and School of Physics and Astronomy, Collaborative Innovation Center for IFSA (CICIFSA), State Key Laboratory of Dark Matter Physics, Shanghai Jiao Tong University, Shanghai, China; 3 Department of Nuclear Engineering and Management, Graduate School of Engineering, The University of Tokyo, Bunkyo-ku, Tokyo, Japan; 4 Photon Science Center, Graduate School of Engineering, The University of Tokyo, Bunkyo-ku, Tokyo, Japan; 5 Research Institute for Photon Science and Laser Technology, The University of Tokyo, Bunkyo-ku, Tokyo, Japan; 6 Institute for Attosecond Laser Facility, The University of Tokyo, Bunkyo-ku, Tokyo, Japan

**Keywords:** Ultrafast photonics, Single photons and quantum effects

## Abstract

Quantitative control and measurement of quantum entanglement are essential for advancing quantum technologies. Photoionization induced by ultrashort laser pulses provides a unique platform for studying entanglement between photoelectrons and residual ions, representing one of the most intriguing quantum phenomena in attosecond physics. Although extensive studies have focused on the coherence properties within either the emitted electrons or the ions individually, the electron-ion entanglement has remained largely unexplored. In this work, we bridge this gap by investigating the resonance-enhanced multiphoton ionization of argon atoms driven by two time-delayed ultrashort ultraviolet pulses. Employing state-of-the-art first-principles multi-electron simulations, we demonstrate the ability to reconstruct and precisely manipulate the purity of electron quantum states through detailed analysis of the photoelectron angular distributions. Our results reveal distinct scattering-phase differences among various electron configurations within the same partial wave channel, providing unequivocal evidence of electron-ion correlation and entanglement. With the fast development of free-electron lasers, this study establishes an experimentally feasible framework for directly controlling quantum entanglement in ultrafast ionization processes, offering new insights and powerful methodologies for exploring complex electron dynamics in many-electron systems.

## Introduction

Quantum entanglement is a hallmark of quantum mechanics and a striking departure from classical physics^1^. Beyond serving as a conceptual cornerstone for understanding quantum theory, entanglement is also recognized as a crucial resource for emerging quantum technologies, including quantum computation, metrology, and entanglement-enhanced imaging^1–8^. In ultrafast physics, entanglement continues to reveal new dimensions of complexity and novel opportunities. Ultrafast physics aims to probe and control electron dynamics on time scales ranging from femtoseconds down to tens or hundreds of attoseconds, pushing the boundaries of the measurement and manipulation of quantum systems^9,10^. With the development of ultrashort laser pulses, a rich suite of imaging and spectroscopic techniques has been developed to visualize and control electron motion in atoms, molecules, and condensed matter systems, such as high harmonic spectroscopy^11–13^, laser-induced electron diffraction^14,15^, photoelectron holography^16–18^, attosecond streaking^19–23^, and attosecond pump-probe spectroscopy^24–26^. Central to these techniques is photoionization, a phenomenon that has long served as a testing ground for quantum mechanics and has played a fundamental role in the development of ultrafast measurement techniques. While the quantum nature of photoionization is well established, our understanding of how entanglement both shapes and emerges from this process remains largely unexplored^9,27^.

In photoionization, entanglement naturally emerges from correlations between the emitted photoelectron and the residual ion. Photoionization of many-electron systems typically leaves the parent ion in an ensemble of symmetry- and energy-allowed quantum states. Consequently, neither the photoelectron nor the ion subsystem individually maintains a pure quantum state, reflecting intrinsic electron-ion entanglement. Recent experimental and theoretical advances have begun illuminating the significance of electron-ion entanglement^28^. Numerical tests of Bell inequalities in strong-field ionization of argon atoms have revealed entanglement signatures between electrons and ions^2^. When multiple ionic states in neon atoms are coupled via Rabi oscillations, entanglement enables the interference of photoelectrons originating from distinct electron shells^29^. In molecular systems, such as ${{\rm{H}}}_{2}$, the degree of entanglement between the photoelectron energy states and the vibrational states of the molecular ion in photoionization can be probed by the dissociation of the ${{\rm{H}}}_{2}^{+}$ ion^30,31^.

Despite these efforts, direct quantitative characterization and control of entanglement in photoionization remain highly challenging. Experimental constraints, such as the necessity to measure incompatible observables, fundamentally hinder practical entanglement characterization. While approaches like KRAKEN^32,33^ offer proof-of-principle demonstrations of density-matrix reconstruction, and hence quantitative entanglement characterization, broadly applicable and experimentally straightforward methods for routine entanglement control are notably lacking. Therefore, strategies leveraging readily measurable observables to simultaneously probe and manipulate electron-ion entanglement are critically needed.

In this work, we introduce an experimentally feasible approach to probe and control electron-ion entanglement by manipulating the orbital angular momentum states of both the ion and electron, with delay-dependent photoelectron angular distributions (PADs) serving as readily accessible observables. Specifically, we explore a $(2+{1}^{{\prime} })$ resonance-enhanced multiphoton ionization (REMPI) process in argon atoms, where the atom is first excited to the 4*p* level using a $\sim 186$ nm femtosecond pump pulse and subsequently ionized by a delayed ultraviolet pulse with a wavelength of $\sim 130$ nm. The adopted laser sources are accessible via tabletop frequency-conversion of the Ti:sapphire laser^34^, high harmonic generation, or modern free-electron lasers (FELs)^25,35^. By varying the time delay between the pulses, we coherently manipulate the superposition of the resulting multi-electron states, thereby inducing controllable changes in the purity of the photoelectron states. These purity variations are directly encoded into the PADs and can therefore be reconstructed. Furthermore, by analyzing the PADs over a range of delays, we uncover unique scattering-phase differences between distinct multi-electron states, directly exposing the underlying electron-ion correlations. The ability to probe and actively control entanglement in ultrafast processes sets the stage for future developments in entanglement-based quantum technologies, advancing the frontiers of ultrafast science and quantum control.

## Results

### Principle of the two-stage scheme

**Fig. 1:**
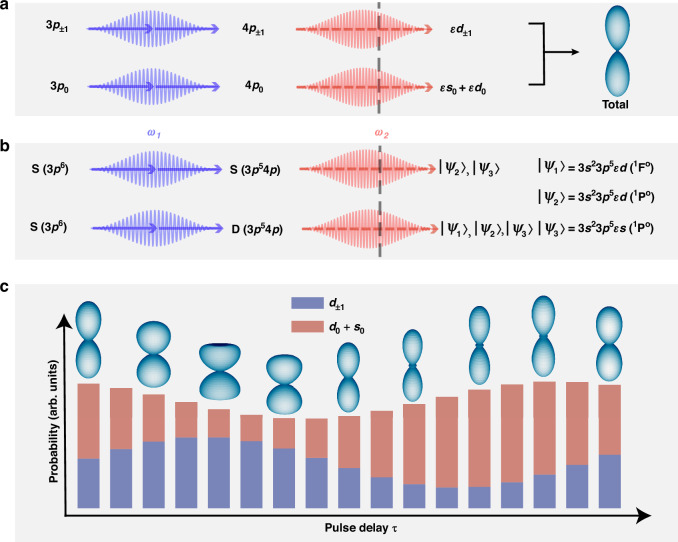
The two-stage ionization scheme applied to argon atoms. **a** Schematic diagram of the two-stage ionization of argon atoms driven by pulses with frequencies ${\omega }_{1}$ and ${\omega }_{2}$ under SAE approximation, with a time delay $\tau$ between the pulses. The dashed gray curve stands for the first ionization threshold. The PAD, combining contributions from the ${m}_{e}=0$ and ${m}_{e}=\pm 1$ orbitals, is shown on the right. **b** Same as **a** but with full consideration of electron-ion coupling. $|{\psi }_{\mathrm{1,2,3}}\rangle$ are the multi-electron states considered. **c** The proportion of the ${m}_{e}=0$ and ${m}_{e}=\pm 1$ states in the mixed state as a function of the pulse delay, obtained from the same calculations as in (**b**). Corresponding PADs are displayed above each bar

Figure 1 illustrates the two-stage scheme utilized in this study. In the first stage, the argon atom is resonantly excited to the $4$*p* state via the absorption of two linearly polarized photons with angular frequency ${\omega }_{1}$. In the second stage, a time-delayed pulse with frequency ${\omega }_{2}$ ionizes the excited atom, ejecting the electron to the continuum states between the first and second ionization thresholds. Here, we neglect spin-orbit coupling. The spin polarizations of the photoelectron and the ion are conserved, and therefore, we can disregard the degree of spin in the following discussion. The residual ion can occupy magnetic quantum states ${m}_{i}=0$ or ${m}_{i}=\pm 1$. The photoelectron with the magnetic quantum number ${m}_{e}$ is always correlated with an ion whose magnetic quantum number satisfies ${m}_{i}=-{m}_{e}$, resulting in an entangled electron-ion state $\left|{m}_{i}\right\rangle \left|{m}_{e}\right\rangle$. With ${C}_{{m}_{e}}$ as the amplitude of such a state, the total wave function of the electron-ion system can then be expanded as

1 $$|\Psi \rangle =\mathop{\sum }\limits_{{m}_{e}}C_{{m}_{e}}|{m}_{i}\rangle |{m}_{e}\rangle$$

Consequently, the photoelectron originating from the outermost *p*-shell resides in a mixed state rather than a pure state. The density operator of the photoelectron, ${\rho }_{e}$, can be derived from the total density operator $\rho =|\Psi \rangle \langle \Psi |$ by tracing out the ionic degrees of freedom as

2 $${\rho }_{e}={{\mathrm{Tr}}}_{i}[\rho ]=\mathop{\sum }\limits_{{m}_{e}}|{C}_{{m}_{e}}{|}^{2}|{m}_{e}\rangle \langle {m}_{e}|$$

The coefficients $|{C}_{{m_e}}{|}^{2}$ govern the photoelectron state purity, which can be tuned by adjusting the inter-pulse delay $\tau$, as demonstrated in subsequent analysis.

Within the single-active-electron (SAE) approximation, ionization from the valence shell orbitals of argon atoms for ${m}_{e}=0$ and ${m}_{e}=\pm 1$ is treated independently. Through two-photon absorption, electrons in the $3{p}_{{m}_{e}}$ orbitals are excited to the $4{p}_{{m}_{e}}$ states. Subsequent absorption of an ${\omega }_{2}$ photon can populate the electron to the continuum ${s}_{0}$, ${d}_{0}$, and ${d}_{\pm 1}$ waves, as illustrated in Fig. 1a. In experiments, where the internal states of the ion are not measured, the photoelectron momentum distribution (PMD) corresponds to an incoherent summation of contributions from the three ionic states. The associated PAD at energy $\varepsilon =2{\omega }_{1}+{\omega }_{2}-{I}_{p}$, where $I_p$ is the first ionization potential of argon atoms, is sketched in Fig. 1a. Although the entanglement between the photoelectron and the ion persists in this formalism, its quantitative characterization becomes inherently incomplete. If the excited-state dissipation is neglected, the weights of the ${m}_{e}=0$ and ${m}_{e}=\pm 1$ partial waves in the mixed state, along with the resulting PAD, remain invariant to time-delay variations.

In the multi-electron picture, the $4p$ level splits into a manifold of substates corresponding to the total angular momentum $L$ of the entire system. As shown in Fig. 1b, substates with $3{s}^{2}3{p}^{5}4p\left({1\atop }{\rm{S}}^{\rm{e}}\right)$ [The term symbol ${2S+1\atop }{L}^{\pi}$ describes the multi-electron state of an atom in the ${LS}$ coupling, where $S$ and $L$ are the total spin and orbital angular momentum, respectively. $L$ is written in spectroscopic notation like ${\rm{S}}$, ${\rm{P}}$, ${\rm{D}}$,..., corresponding to $L=\mathrm{0,1,2},\ldots$. $\pi ={\rm{o}}({\rm{e}})$ denotes the odd (even) parity, which is determined by ${(-1)}^{{\sum }_{i}{l}_{i}}$ (1 corresponds to odd parity and 0 corresponds to even parity), where ${l}_{i}$ is the orbital angular momentum of each electron. The spin multiplicity is determined by $2S+1$. Note that this notation also applies for continuum states.] and $3{s}^{2}3{p}^{5}4p\left({1 \atop}{\rm{D}}^{\rm{e}}\right)$ are accessible via two-photon transitions from the $3{s}^{2}3{p}^{6}\left({1\atop }{\rm{S}}^{\rm{e}}\right)$ ground state, inducing a quantum beat. Although the spin-orbit coupling effect can further split these states into five sublevels, the fine structures can be neglected due to the relatively broad spectral width of the applied pulses. The energy differences between the ground state and the two resonant states are $13.19$ eV $({1\atop }{\rm{D}}^{\rm{e}})$ and $13.38$ eV $\left(^1{\rm{S}}^{\rm{e}}\right)$ , respectively, giving rise to a term splitting of $0.19$ eV. The coherent superposition of the two resonant states can lead to a quantum beat with an oscillation period of $21.77$ fs.

For electrons reaching the same final energy after absorbing one photon from the second pulse, the delay $\tau$ is expected to play a crucial role in governing PADs by modulating the phase difference between different ionization pathways. It should be mentioned that the ionization process we study is at a femtosecond timescale, which is far smaller than the typical picosecond lifetime of the $4p$ excited levels. As a result, the decoherence during this process can be safely neglected. We focus on three dominant resonance-enhanced pathways, which correspond to transitions leading to three different final states $3{s}^{2}3{p}^{5}\varepsilon d\left({1\atop }{\rm{F}}^{\rm{o}}\right)$, $3{s}^{2}3{p}^{5}\varepsilon d\left({1\atop }{\rm{P}}^{\rm{o}}\right)$, and $3{s}^{2}3{p}^{5}\varepsilon s\left({1\atop }{\rm{P}}^{\rm{o}}\right)$. By tuning $\tau$, it is possible to manipulate the ratio between the ${m}_{e}=0$ and ${m}_{e}=\pm 1$ components, producing oscillatory patterns in the PADs. Figure 1c illustrates these effects, showing the relative contributions of the ${m}_{e}=0$ and ${m}_{e}=\pm 1$ components and their corresponding PADs. This reveals how the entanglement between the photoion and photoelectron manifests in the PADs.

### Multi-pathway interference

We first provide an explicit derivation of how multi-pathway interference governs the probabilities of photoelectron partial-wave populations and consequently shapes the PADs. To analyze the interacting electron-ion system, the coupled representation provides a natural framework. In this representation, angular states are expressed as $\left|{LM}{l}_{i}{l}_{e}\right\rangle$, where $L$, ${l}_{i}$, and ${l}_{e}$ denote the angular momenta of the whole system, the ion, and the emitted electron, respectively. Their corresponding magnetic quantum numbers are $M$, ${m}_{i}$, and ${m}_{e}$, satisfying $M={m}_{i}+{m}_{e}$. Using this notation, the final states of interest, namely, $3{s}^{2}3{p}^{5}\varepsilon d\left({1\atop }{\rm{F}}^{\rm{o}}\right)$, $3{s}^{2}3{p}^{5}\varepsilon d\left({1\atop }{\rm{P}}^{\rm{o}}\right)$, and $3{s}^{2}3{p}^{5}\varepsilon s\left({1\atop }{\rm{P}}^{\rm{o}}\right)$, are relabeled as $\left|3012\right\rangle$, $\left|1012\right\rangle$, and $\left|1010\right\rangle$, respectively. The transformation between coupled and decoupled representations is mediated by Clebsch-Gordan (CG) coefficients as

3 $$\left|{LM}{l}_{i}{l}_{e}\right\rangle =\sum {C}_{{l}_{i},{m}_{i},{l}_{e},{m}_{e}}^{L,M}\left|{l}_{i}{m}_{i}\right\rangle \left|{l}_{e}{m}_{e}\right\rangle$$

Expanding the coupled states into decoupled basis states yields

4 $$\begin{array}{c}\left|3012\right\rangle \end{array}=\sqrt{\frac{1}{5}}\left|{p}_{-1}\right\rangle \left|{d}_{1}\right\rangle +\sqrt{\frac{3}{5}}\left|{p}_{0}\right\rangle \left|{d}_{0}\right\rangle +\sqrt{\frac{1}{5}}\left|{p}_{1}\right\rangle \left|{d}_{-1}\right\rangle$$

5 $$\left|1012\right\rangle =\sqrt{\frac{3}{10}}\left|{p}_{-1}\right\rangle \left|{d}_{1}\right\rangle -\sqrt{\frac{2}{5}}\left|{p}_{0}\right\rangle \left|{d}_{0}\right\rangle +\sqrt{\frac{3}{10}}\left|{p}_{1}\right\rangle \left|{d}_{-1}\right\rangle$$

6 $$\left|1010\right\rangle =|{p}_{0}\rangle |{s}_{0}\rangle$$

For conciseness, we define $\left|3012\right\rangle =\left|{\psi }_{1}\right\rangle$, $\left|1012\right\rangle =\left|{\psi }_{2}\right\rangle$, and $\left|1010\right\rangle =\left|{\psi }_{3}\right\rangle$. The state of the total electron-ion system is then described by

7 $$\left|\Psi \right\rangle =\mathop{\sum }\limits_{j=1}^{3}{C}_{{\psi }_{j}}\left|{\psi }_{j}\right\rangle$$

where ${C}_{{\psi }_{j}}$ demonstrates the amplitude of state $\left|{\psi }_{j}\right\rangle$. Substituting Eqs. (4-6) into Eq. (7) recovers Eq. (1). The partial wave probabilities of the photoelectron are determined by the diagonal entries of the density matrix as ${M}_{{l}_{e}{m}_{e}}=\left\langle {l}_{e}{m}_{e}\left|{\rho }_{e}\right|{l}_{e}{m}_{e}\right\rangle$, which are explicitly given by

8 $$\begin{array}{c}{M}_{{s}_{0}}\end{array}={\left|{C}_{{\psi }_{3}}\right|}^{2}$$

9 $${M}_{{d}_{1}}={\left|\sqrt{\frac{1}{5}}{C}_{{\psi }_{1}}+\sqrt{\frac{3}{10}}{C}_{{\psi }_{2}}\right|}^{2}$$

10 $${M}_{{d}_{0}}={\left|\sqrt{\frac{3}{5}}{C}_{{\psi }_{1}}-\sqrt{\frac{2}{5}}{C}_{{\psi }_{2}}\right|}^{2}$$

These equations demonstrate the connections between the multi-path interference and the partial-wave populations. The values of partial-wave populations will determine the PADs, which can be probed experimentally. The complete angular distribution is expressed as a two-dimensional function of the polar angle $\Omega =\left(\theta ,\varphi \right)$. Analogous to Eqs. (8-10), it is derived from the diagonal entries of the density operator, $I\left(\Omega \right)=\left\langle \Omega \left|{\rho }_{e}\right|\Omega \right\rangle$. Explicitly, it can be expressed as

11 $$I(\Omega )=\mathop{\sum }\limits_{{l}_{e}{m}_{e},{l}_{e}^{{\prime} }{m}_{e}^{{\prime} }}{Y}_{{l}_{e}{m}_{e}}(\Omega )\langle {l}_{e}{m}_{e}|{\rho }_{e}|{l}_{e}^{{\prime} }{m}_{e}^{{\prime} }\rangle {Y}_{{l}_{e}^{{\prime} }{m}_{e}^{{\prime} }}^{* }(\Omega )$$

where ${Y}_{{l}_{e}{m}_{e}}\left(\Omega \right)$ are the spherical harmonics. The contributions from different ${m}_{e}$ states to the PADs are incoherent since $\left\langle {l}_{e}{m}_{e}\left|{\rho }_{e}\right|{l}_{e}^{{\prime} }{m}_{e}^{{\prime} }\right\rangle \ne 0$ only when ${m}_{e}={m}_{e}^{{\prime} }$, hence $I\left(\Omega \right)$ is independent of the azimuthal angle $\varphi$ when only linearly polarized fields are applied, consistent with the cylindrical symmetry of the system. Thus, we simplify the notation to $I\left(\theta \right)$.

To analyze the PADs more quantitatively, we use the anisotropy parameters^29,36,37^. Providing that the two pulses do not overlap in the time domain, non-resonant ionization pathways leading to $f$ and higher angular momentum states are negligible. Our first-principles calculations have shown that the higher partial waves are more than $10^3$ times weaker than $\left({s}_{0},{d}_{0},{d}_{\pm 1}\right)$ in the energy range we are interested in. Therefore, we expand the delay-dependent PAD via the leading order Legendre polynomials as

12 $$I\left(\theta \right)=\mathop{\sum }\limits_{n=0,2,4}{b}_{n}{P}_{n}\left(\cos \theta \right)$$

with

13 $${b}_{n}=\mathop{\sum }\limits_{{m}_{e}}\mathop{\sum }\limits_{{l}_{e}{l}_{e}^{{\prime} }}\frac{{(-1)}^{{m}_{e}}\sqrt{\left(2{l}_{e}+1\right)\left(2{l}_{e}^{{\prime} }+1\right)}}{4\pi }\left\langle {l}_{e}0{l}_{e}^{{\prime} }0|n0\right\rangle \left\langle {l}_{e}{m}_{e}{l}_{e}^{{\prime} }\left(-{m}_{e}\right)|n0\right\rangle \left\langle {l}_{e}{m}_{e}\left|{\rho }_{e}\right|{l}_{e}^{{\prime} }{m}_{e}\right\rangle$$

where $\left\langle {l}_{e}0{l}_{e}^{{\prime} }0|n0\right\rangle$ and $\left\langle {l}_{e}{m}_{e}{l}_{e}^{{\prime} }\left(-{m}_{e}\right)|n0\right\rangle$ are the CG coefficients. The anisotropy parameters ${b}_{n}$ relate to the conventional anisotropy parameters ${\beta }_{n}$^29,36,37^ with ${\beta }_{n}={b}_{n}/{b}_{0}$ and ${b}_{n}$ are all real numbers. For the $\left(2+{1}^{{\prime} }\right)$ REMPI via the *p* resonant states in argon atoms, the ${m}_{e}=0$ electron can be ejected to ${s}_{0}$ and ${d}_{0}$ waves. Assuming that the complex amplitudes of the partial waves $\left|{l}_{e}{m}_{e}\right\rangle$ are denoted as ${C}_{{l}_{e}{m}_{e}}$, the PAD for the ${m}_{e}=0$ electron can be expressed as

14 $${I}_{0}\left(\theta \right)={\left|{C}_{{s}_{0}}{Y}_{00}\left(\theta \right)+{C}_{{d}_{0}}{Y}_{20}\left(\theta \right)\right|}^{2}=\frac{1}{4\pi }{M}_{{s}_{0}}+\frac{\sqrt{5}}{2\pi }{\mathrm{Re}}\left[{C}_{{s}_{0}}^{* }{C}_{{d}_{0}}\right]{P}_{2}\left(\cos \theta \right)+\frac{5}{4\pi }\left[\frac{18}{35}{P}_{4}\left(\cos \theta \right)+\frac{2}{7}{P}_{2}\left(\cos \theta \right)+\frac{1}{5}\right]{M}_{{d}_{0}}$$

with ${Y}_{l0}\left(\theta \right)=\sqrt{\frac{2l+1}{4\pi }}{P}_{l}\left(\cos \theta \right)$ and ${M}_{{l}_{e}{m}_{e}}=|{C}_{{l}_{e}{m}_{e}}{|}^{2}$. Similarly, since the ${m}_{e}=\pm 1$ electron can only be ejected to ${d}_{\pm 1}$ waves, the PAD for the ${m}_{e}=\pm 1$ orbitals can be obtained as

15 $$\begin{array}{lll}{I}_{\pm 1}(\theta )&=&|{C}_{{d}_{\pm 1}}{Y}_{2\pm 1}(\theta ,\varphi ){|}^{2}\\&&=\frac{1}{\pi }\left[-\frac{3}{7}{P}_{4}\left(\cos \theta \right)+\frac{5}{28}{P}_{2}\left(\cos \theta \right)+\frac{1}{4}\right]{M}_{{d}_{\pm 1}}\end{array}$$

Considering the contributions from the ${m}_{e}=\pm 1$ orbitals are the same, the total PAD can be calculated as $I\left(\theta \right)={I}_{0}\left(\theta \right)+2{I}_{1}\left(\theta \right)$. By projecting the PAD onto the Legendre polynomials based on Eq. (12), we can obtain that

16 $$\begin{array}{c}{b}_{0}\end{array}=\frac{1}{4\pi }({M}_{{s}_{0}}+{M}_{{d}_{0}}+2{M}_{{d}_{1}})$$

17 $${b}_{2}=\frac{5}{14\pi }\left({M}_{{d}_{0}}+{M}_{{d}_{1}}\right)+\frac{\sqrt{5}}{2\pi }{\mathrm{Re}}\left[{C}_{{s}_{0}}^{* }{C}_{{d}_{0}}\right]$$

18 $${b}_{4}=\frac{3}{14\pi }\left(3{M}_{{d}_{0}}-4{M}_{{d}_{1}}\right)$$

The anisotropy parameters ${b}_{n}$, which depend on the time delay $\tau$, quantify the angular modulation of the PAD and reflect the interference between different partial waves. By tuning $\tau$, the phase relationships between resonant pathways are modified, thereby governing the observed PAD. This enables a direct probe of the entanglement dynamics.

### Delay-dependent PADs from first-principles multi-electron simulations

**Fig. 2:**
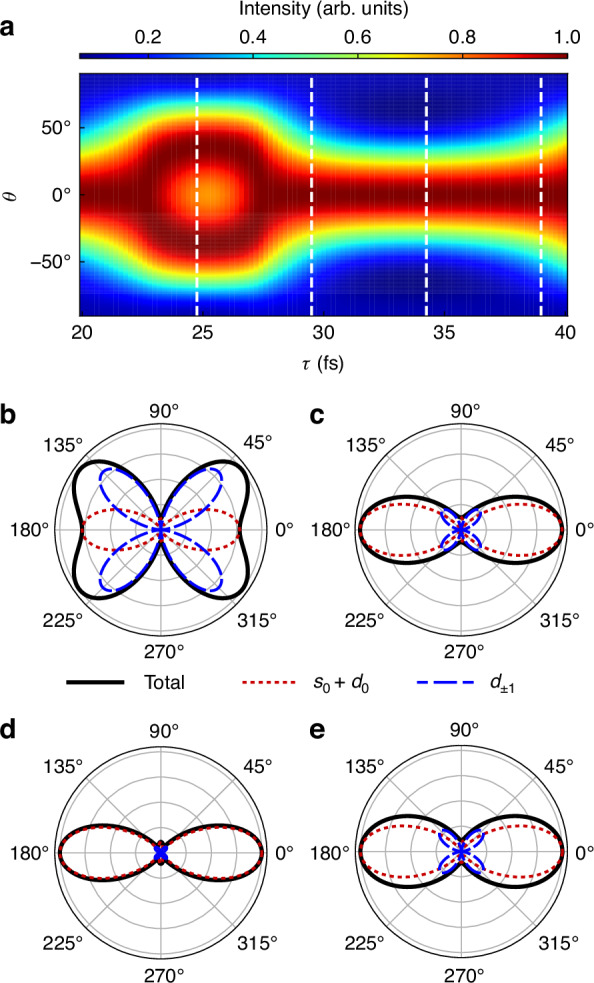
Delay-dependent photoelectron angular distributions. **a** PADs at energy $\varepsilon =2{\omega }_{1}+{\omega }_{2}-{I}_{p}$ as functions of the emission angle $\theta$ and the time delay *τ* between the first pulse (${\omega }_{1}=6.52$ eV) and the second pulse (${\omega }_{2}=9.52$ eV). The PAD intensities are normalized independently at each *τ*. The white dashed lines mark the PADs at the delays of $24.75$, $29.5$, $34.25$, and $39$ fs. **b**–**e** PADs at delays of $24.75$, $29.5$, $34.25$, and $39$ fs. Black, red, and blue lines denote the total signals and contributions from ${m}_{e}=0$ and ${m}_{e}=\pm 1$, respectively

Using the multi-configurational time-dependent Hartree-Fock (MCTDHF) method^38–41^, along with the R-matrix with time-dependence (RMT) method^42^ as a cross check, we perform simulations of $(2+{1}^{{\prime} })$ REMPI process of argon atoms (see materials and methods for details). The resulting delay-dependent PADs are shown in Fig. 2a, which are obtained by integrating over the peak signal in the vicinity of $\varepsilon =2{\omega }_{1}+{\omega }_{2}-{I}_{p}$ and normalized independently at each $\tau$. Significant delay-dependent modulations can be observed in the PADs. The PAD maximum initially appears at ${0}^{\circ }$ at $\tau =20$ fs. Then it gradually shifts to ${40}^{\circ }$ at $\tau =24$ fs and returns to ${0}^{\circ }$ at $\tau =30$ fs. Simultaneously, the PAD minimum gradually changes from ${90}^{\circ }$ to $6{0}^{\circ }$. This delay-dependent evolution manifests the quantum beat between the resonant states ^1^S^e^ and ^1^D^e^.

Figure 2b–e display the PADs at specific delays of $\mathrm{24.75,29.5,34.25}$, and $39$ fs, highlighting distinct angular features. The four panels correspond to the lineouts at the delays marked by vertical white dashed lines in Fig. 2a. In addition to the total PADs (black solid lines), we show the separate contributions from ${s}_{0}+{d}_{0}$ (red dotted lines) and ${d}_{\pm 1}$ partial waves (blue dashed lines), where the ${d}_{\pm 1}$ term collects the contributions from ${d}_{1}$ and ${d}_{-1}$. In essence, the change of the PADs is a reflection of the delay-varying proportions of the ${d}_{\pm 1}$ and ${d}_{0}$ waves. For instance, in Fig. 2b and d, the total PADs predominantly reflect ${d}_{\pm 1}$- or ${d}_{0}$- wave characteristics, respectively. For the cases presented in Fig. 2c and e, where the ${m}_{e}=0$ and ${m}_{e}=\pm 1$ components exhibit comparable contributions, the resulting PAD demonstrates marked deviations from the characteristic patterns of individual components, manifesting a unique hybrid structure. At $\tau =39$ fs, the PADs initiate a new cycle, signaling the periodic nature of the dynamics. The determined period of approximately $19$ fs aligns with the quantum beat period derived from the resonant states with an energy splitting of $0.22$ eV, underscoring the strong connection between the temporal evolution of PADs and the underlying coherent processes. This periodic modulation demonstrates that coherent control over the PADs can be achieved by varying the pulse delay, a hallmark of the two-stage ionization scheme.

Within expectation, no delay-dependent modulation emerges under the SAE approximation (not shown). In the absence of the quantum beat, the PAD derived under the SAE approximation remains static over time and closely resembles a ${d}_{0}$ wave, consistent with the intuitive expectation that ionization from the ${m}_{e}=0$ orbital dominates in a linearly polarized field. However, the multi-electron calculations reveal starkly different dynamics, with delay-dependent modulations driven by coherence and entanglement effects. These unique features emphasize the limitations of the SAE model and the necessity of multi-electron approaches for capturing the full complexity of the system. This contrast highlights the essential role of quantum coherence and entanglement in the observed dynamics, providing a deeper understanding of the interplay between resonant states and their contributions to PADs.

### Reconstruction of photoelectron purity

The two-stage scheme effectively achieves ultrafast control of the photoelectron purity, which is encoded in the PADs. Given the photoelectron density operator ${\rho }_{e}$, the purity is defined as

19 $${P}_{e}=\mathrm{Tr}\left[{\rho }_{e}^{2}\right]$$

The trace operation ensures that ${P}_{e}$ is independent of the choice of representation. In terms of the partial-wave probabilities, ${P}_{e}$ can be written as

20 $${P}_{e}={\mathrm{Tr}}\left[{\rho }_{e}^{2}\right]=\frac{{\left({M}_{{s}_{0}}+{M}_{{d}_{0}}\right)}^{2}+2{M}_{{d}_{1}}^{2}}{{\left({M}_{{s}_{0}}+{M}_{{d}_{0}}+2{M}_{{d}_{1}}\right)}^{2}}$$

which depends on the emission energy $\varepsilon$ and the time delay *τ* (see supplementary information). The purity of a mixed state is always less than $1$. Values close to $1$ indicate strong dominance of a specific ${m}_{e}$ channel in the final state. This indicates that the entanglement between the ion and electron is low. On the other hand, the minimum value of $1/3$ corresponds to a maximally mixed state. This minimum occurs when the populations of the ${m}_{e}=0$ and ${m}_{e}=\pm 1$ states are equal, i.e., $|{C}_{0}{|}^{2}=|{C}_{1}{|}^{2}=|{C}_{-1}{|}^{2}$. This also reflects that the ion and electron are in the maximally entangled state. We note here that in our framework, the state of the whole system is pure, so the electron’s reduced-state purity serves as a direct and standard measure for electron-ion entanglement. The commonly used I-concurrence in this setting is just a one-to-one monotonic function of the purity, given by $I=\sqrt{2(1-{P}_{e})}$. Throughout the work, we consistently use purity as a measure of the entanglement. It should be mentioned that, since we mainly focus on electron-ion entanglement in the degree of angular momentum, we do not further distinguish the spin polarization for the electron or the ion. As a result, signals with the same ${m}_{e}$ but different spin polarization are summed incoherently.

The delay-dependent PADs only provide qualitative insights into the purity variations. To gain a quantitative assessment, we need to analyze the anisotropy parameters, which can be extracted by projecting the PADs onto the Legendre polynomials based on Eq. (12). As the PADs depend on $\tau$, we obtain a series of anisotropy parameters as functions of $\tau$. According to Eq. (20), the evaluation of the electronic-state purity ${P}_{e}$ requires the partial-wave intensities ${M}_{{s}_{0}}$, ${M}_{{d}_{0}}$, and ${M}_{{d}_{1}}$. However, with the anisotropy parameters obtained from the PADs alone, the partial-wave intensities are unable to be directly retrieved by solving Eqs. (16-18), since there are three equations but four unknowns. This notorious difficulty arises from the lack of phase information in the PADs, which is inevitable when ${d}_{\pm 1}$ is not strictly zero. To overcome it, we solve Eqs. (16-18) at some $\tau$ by setting $\zeta =\cos \left[{\rm{\arg }}({C}_{{d}_{0}}/{C}_{{s}_{0}})\right]$ to a given value. By scanning $\zeta$ across its physically admissible range at this $\tau$, we obtain a mapping from $(\tau ,\zeta )$ to the purity ${P}_{e}$, denoted by ${P}_{e}(\tau ,\zeta )$. The true value of the purity should be ${P}_{e}(\tau ,\zeta (\tau ))$ where $\zeta (\tau )$ is the true value of the unknown $\cos \left[{\rm{\arg }}({C}_{{d}_{0}}/{C}_{{s}_{0}})\right]$. To predict the range of possible values of the purity, we calculate the mean values ${\bar{P}}_{e}$ as well as the standard deviations ${S}_{{P}_{e}}$ via

21 $${\bar{P}}_{e}=\int {P}_{e}\left(\tau ,\zeta \right)d\zeta /\int d\zeta$$

22 $${S}_{{P}_{e}}=\sqrt{\int {\left[{P}_{e}(\tau ,\zeta )-{\bar{P}}_{e}\right]}^{2}d\zeta /\int d\zeta }$$

**Fig. 3:**
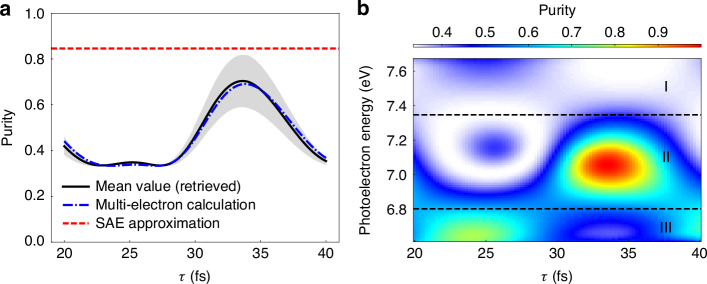
Reconstruction of photoelectron purity. **a** Reconstructed photoelectron purity inferred from PADs. The black solid line and the gray shaded region show the mean values $\bar{{P}_{e}}$ and the standard deviations ${S}_{{P}_{e}}$ of the reconstructed results. The blue and red dashed lines correspond to purity directly derived from the multi-electron calculations and from the calculations under the SAE approximation, respectively. **b** Energy-resolved purity from multi-electron simulations as a function of pulse delay $\tau$. The energy range in the vicinity of $\varepsilon =2{\omega }_{1}+{\omega }_{2}-{I}_{p}$ is displayed. Three distinct regimes (I–III) emerge, characterized by different delay-dependent modulation patterns

For some $(\tau ,\zeta)$ pairs, there are no strict solutions for ${P}_{e}$. In such cases, we find the approximate solutions that deviate least from Eqs. (16-18) while maintaining the consistency of the obtained ${M}_{{s}_{0}},{M}_{{d}_{0}}$, and ${M}_{{d}_{1}}$ with respect to $\tau$. This approach enables quantitative purity estimation solely from PADs. As shown in Fig. 3a, the reconstructed purity (black solid line showing $\bar{{P}_{e}}$, with gray shading indicating ${S}_{{P}_{e}}$) aligns closely with direct multi-electron calculations (blue dashed line), validating our methodology.

Two local purity maxima occur at delays of $25$ and $33$ fs, corresponding to the dominance of the ${d}_{\pm 1}$ and ${d}_{0}$ waves, respectively. In an idealized scenario where the ${d}_{\pm 1}$ or ${d}_{0}$ waves completely dominate, the purity should be $0.5$ ($|{C}_{0}{|}^{2}=0$, $|{C}_{\pm 1}{|}^{2}=1/2$) or 1 ($|{C}_{0}{|}^{2}=1$, $|{C}_{\pm 1}{|}^{2}=0$). However, our calculated maxima are reduced to $0.34$ and $0.69$, indicating the non-negligible contributions from the ${m}_{e}=0$ or ${m}_{e}=\pm 1$ orbitals, respectively. At delays of $18$ and $28$ fs, the purity reaches the minima of $0.33$ due to equal contributions from different ${m}_{e}$ channels, which aligns with our previous expectation. In great contrast, under the SAE approximation, the photoionization signal predominantly originates from the ${m}_{e}=0$ orbital, predicting a delay-invariant high purity. The result is sketched by the red dashed line in Fig. 3a, which differs sharply from the results of the multi-electron calculations, where the orbital dominance varies with time. This discrepancy underscores the critical role of electron-ion entanglement in REMPI of multi-electron systems.

The systematically higher purity in SAE simulations compared to multi-electron results initially appears counterintuitive. To understand this phenomenon and the difference between the observed purity maxima and the idealized predictions, we analyze the photoelectron-energy-resolved purity. Figure 3b shows the photoelectron purity from multi-electron calculations near $\varepsilon =2{\omega }_{1}+{\omega }_{2}-{I}_{p}$. The data reveals three energy regions, exhibiting distinct variations of purity as a function of pulse delay $\tau$. The central region (region II) features the most pronounced ionization peak, with two local maxima at the same $\tau$ as the total purity. The two maxima reach almost $0.5$ and $1$, respectively, indicating the complete dominance of ${d}_{\pm 1}$ or ${d}_{0}$ waves at the two delays. In regions I and III, the purity variations show completely different behaviors. Energy-resolved partial-wave analysis indicates that at $\tau =25$ fs, the ${d}_{0}$ wave’s peak splits into two peaks located in regions I and III. This allows ${m}_{e}=0$ contributions to surpass those from the ${m}_{e}=\pm 1$ orbitals near the edges of the peak, resulting in relatively high purity. Conversely, at $\tau =33$ fs, the peaks of the ${d}_{\pm 1}$ waves shift outward, balancing the ${d}_{0}$-wave contributions at the edges and creating observable white and blue areas in regions I and III at this delay. Further discussions of these features are provided in the supplementary information.

As a result, if we combine the three regions together, the dominance of the ${d}_{\pm 1}$ (${d}_{0}$) wave at $\tau =25\,(33)$ fs is weakened, leading to the suppressed purity in Fig. 3a. The splitting of a certain partial wave primarily stems from the slight energy offset between the two resonant states, resulting in subtle deviations in the photoelectron energy of the corresponding REMPI signals. With a large overlapped photoelectron energy region under the applied laser fields, the observed ionization peak emerges from the coherent and incoherent summation of signals from different resonant states. Notably, while the total signal manifests as a single peak, energy-resolved partial-wave analysis indicates that ${d}_{\pm 1}$ and ${d}_{0}$ waves alternate between constructive and destructive interference within the ionization peak. The constructively interfering wave dominates the main peak, while the others split into two secondary peaks at both edges, producing the three distinct regions mentioned above. The SAE calculations, which lack the two resonant states, fail to capture the distinct structures of different partial waves in the ionization peak, leading to a comparably high dominance of a single partial wave.

### Phase difference between multi-electron states

**Fig. 4:**
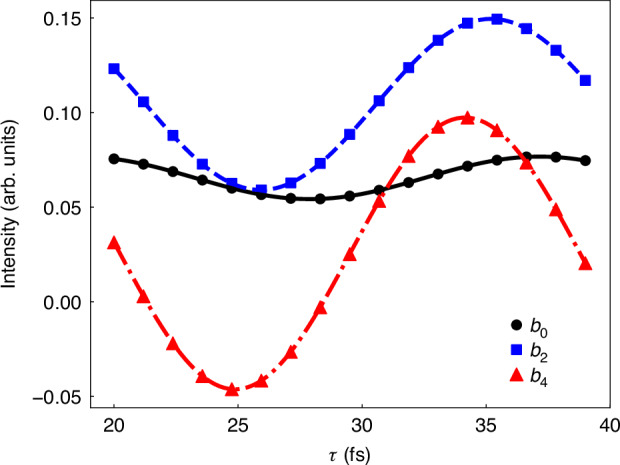
The values of different anisotropy parameters as a function of the pulse delay. The dots are the extracted values from the PADs integrated over the ionization peak energy range, while the curves are the fittings of the dots

The extracted anisotropy parameters from the PADs based on Eq. (12) are displayed by the dots in Fig. 4. We can find that they exhibit oscillations as a function of the pulse delay $\tau$. Fitting these oscillations using the function

23 $$f\left(\tau \right)=G\cos \left(\Delta E\tau +\gamma \right)+H$$

yields $\Delta E=0.223$ eV, corresponding to an oscillation period of $18.55$ fs. This fitting result agrees well with the term splitting of the two resonant states in our calculations (see Materials and Methods). The fittings of the anisotropy parameters are demonstrated by the corresponding curves in Fig. 4. While the extracted energy difference $\Delta E$ aligns with the resonant states, the extracted phase $\gamma$ requires further interpretation. To establish a quantitative link between the quantum beat and the oscillatory PADs, we derive the anisotropy parameters explicitly as a function of the pulse delay $\tau$. This requires evaluating the phases of different states $\left|{\psi }_{i}\right\rangle$ explicitly, which comprise four components: (i) the scattering phase shift of the final state, ${\eta }_{i}$, (ii) the dynamic phase accumulated on the resonant states, $-{E}_{{\rm{S}}/{\rm{D}}}\tau$, (iii) the centrifugal potential phase, $-l\pi /2$, and (iv) an additional phase arising from resonant transitions and laser fields, ${\chi }_{{\rm{S}}/{\rm{D}}}$^36,43,44^. Here, ${E}_{{\rm{S}}\left({\rm{D}}\right)}$ and ${\chi }_{{\rm{S}}\left({\rm{D}}\right)}$ represent the energy of the ^1^S^e^ (^1^D^e^) state and the fourth phase term mentioned above for ionization pathways via the ^1^S^e^ (^1^D^e^) state, respectively. Owing to the selection rule of total angular momentum, $\left|{\psi }_{1}\right\rangle$ can only be ionized from the ^1^D^e^ state. To express the complex amplitude into its absolute value and phase term, we introduce the real amplitude ${\alpha }_{1}^{{\rm{D}}}$, and write the complex amplitude of $\left|{\psi }_{1}\right\rangle$ as

24 $${{\rm{C}}}_{{\psi }_{1}}={{\rm{e}}}^{i\left({\eta }_{1}-\pi \right)}{\alpha }_{1}^{{\rm{D}}}{{\rm{e}}}^{i\left({\chi }_{\mathrm{D}}-{E}_{\mathrm{D}}\tau \right)}$$

Similarly, for $\left|{\psi }_{2}\right\rangle$ and $\left|{\psi }_{3}\right\rangle$, which involve resonant enhancement from both ^1^S^e^ and ^1^D^e^ states, we introduce coefficients ${{\rm{\alpha }}}_{\mathrm{2,3}}^{\mathrm{S}}$ and ${{\rm{\alpha }}}_{\mathrm{2,3}}^{\mathrm{D}}$ to distinguish their contributions. The complex amplitudes of $\left|{\psi }_{2}\right\rangle$ and $\left|{\psi }_{3}\right\rangle$ are given by

25 $${C}_{{\psi }_{2}}={{\rm{e}}}^{i\left({\eta }_{2}-\pi \right)}\left[{\alpha }_{2}^{{\rm{D}}}{{\rm{e}}}^{i\left({\chi }_{{\rm{D}}}-{E}_{{\rm{D}}}\tau \right)}+{\alpha }_{2}^{{\rm{S}}}{{\rm{e}}}^{i\left({\chi }_{{\rm{S}}}-{E}_{{\rm{S}}}\tau \right)}\right]$$

26 $${C}_{{\psi }_{3}}={{\rm{e}}}^{i{\eta }_{3}}\left[{\alpha }_{3}^{{\rm{D}}}{{\rm{e}}}^{i\left({\chi }_{{\rm{D}}}-{E}_{{\rm{D}}}\tau \right)}+{\alpha }_{3}^{{\rm{S}}}{{\rm{e}}}^{i\left({\chi }_{{\rm{S}}}-{E}_{{\rm{S}}}\tau \right)}\right]$$

Using Eqs. (8-10) and (16-18) the partial-wave probabilities are calculated and, subsequently, ${b}_{0}$ and ${b}_{4}$ are written as

27 $$\begin{array}{ccc}{b}_{0}\left(\tau \right) & = & \dfrac{1}{4\pi }\left[{M}_{{s}_{0}}\left(\tau \right)+{M}_{{d}_{0}}\left(\tau \right)+2{M}_{{d}_{1}}\left(\tau \right)\right]\\ & = & {Q}_{{b}_{0}}+\dfrac{1}{2\pi }\left({\alpha }_{2}^{{\rm{D}}}{\alpha }_{2}^{{\rm{S}}}+{\alpha }_{3}^{{\rm{D}}}{\alpha }_{3}^{{\rm{S}}}\right)\cos (\Delta E\tau +{\chi }_{{\rm{DS}}})\end{array}$$

28 $$\begin{array}{ccc}{b}_{4}\left(\tau \right) & = & \dfrac{3}{14\pi }\left[3{M}_{{d}_{0}}\left(\tau \right)-4{M}_{{d}_{1}}\left(\tau \right)\right]\\ & = & {Q}_{{b}_{4}}-\dfrac{3\sqrt{6}}{7\pi }{\alpha }_{1}^{{\rm{D}}}{\alpha }_{2}^{{\rm{S}}}\cos (\Delta E\tau +{\chi }_{{\rm{DS}}}+{\eta }_{12})\end{array}$$

where $\Delta E={E}_{\mathrm{S}}-{E}_{\mathrm{D}}$, ${\chi }_{\mathrm{DS}}={\chi }_{\mathrm{D}}-{\chi }_{\mathrm{S}}$, and ${\eta }_{{ij}}={\eta }_{i}-{\eta }_{j}$. Constants ${Q}_{{b}_{0}}$ and ${Q}_{{b}_{4}}$ encapsulate non-oscillatory terms (see supplementary information).

Fitting ${b}_{0}$ reveals ${\chi }_{\mathrm{DS}}=-0.026$, which is attributed to the action of different intermediate resonant states. Previous studies^36,43,44^ suggested that the phase contributed by the resonant transition depends on the pulse width rather than the specific resonant state, justifying that ${\chi }_{\mathrm{DS}}\approx 0$.

On the other hand, the fitting of ${b}_{4}$ gives rise to ${\eta }_{12}+{\chi }_{\mathrm{DS}}$. The subtraction of ${\chi }_{\mathrm{DS}}$ from this value yields ${\eta }_{12}=-2.119$, reflecting the scattering phase difference between $|{\psi }_{1}\rangle$ and $|{\psi }_{2}\rangle$. ${\eta }_{i}$ in multi-electron systems is composed of the Coulomb phase shift ${\sigma }_{i}$ and a phase from the short-range potential, ${\delta }_{i}$. For $|{\psi }_{1}\rangle$ and $|{\psi }_{2}\rangle$, their partial waves are both $d$ waves, and therefore their Coulomb phase shifts, ${\sigma }_{1}={\sigma }_{2}$. As a result, the phase difference arises primarily from the short-range potential, namely, ${\delta }_{1}-{\delta }_{2}={\eta }_{12}=-2.119$. This result reveals the different short-range interactions in each $|{\psi }_{i}\rangle$, reflecting different ways of the angular momentum coupling between the photoelectron and the ion.

## Discussion

The control and measurement of electron-ion entanglement demonstrated in this study offer a compelling advancement in the broader context of attosecond and ultrafast physics. A distinctive feature of our approach lies in angular momentum coupling among intermediate multi-electron states, allowing for direct access to underlying electron-ion correlations and entanglement through experimentally measurable observables, i.e., PADs. This represents a substantial conceptual step beyond prior methodologies, which often struggled to balance theoretical rigor and experimental feasibility.

A notable outcome of our study is the clear divergence between predictions from the multi-electron approach and the SAE approximation. The SAE framework, conventionally prevalent due to computational simplicity, inherently neglects electron-ion entanglement and thus predicts invariant PAD structures and photoelectron purity. Our results reveal that SAE fails to capture even the qualitative aspects of purity modulation that arise naturally when multi-electron interactions are rigorously treated. This discrepancy not only underscores the inadequacies of simpler models but also reinforces the importance of fully correlated electronic simulations in accurately interpreting attosecond-scale phenomena. Consequently, our findings advocate strongly for shifting future theoretical and experimental research toward comprehensive multi-electron frameworks to reliably interpret subtle quantum effects associated with electron-ion entanglement.

Furthermore, our development of a quantitative method for reconstructing electron purity from anisotropy parameters extracted directly from PADs addresses a longstanding challenge, specifically, the experimental difficulty of accessing quantum entanglement from observables without complete phase information. The current multifragment coincidence detection techniques, such as cold-target recoil ion momentum spectroscopy (COLTRIMS)^45^, are still unable to distinguish the different hole states of the ion entangled with the photoelectrons. Other existing approaches, such as KRAKEN and related quantum tomography techniques^32,33^, demonstrated proof-of-concept capabilities to study entanglement. Their broader adoption has been hindered by experimental complexity and stringent measurement requirements. Our reconstruction strategy, utilizing readily available angular distribution data, significantly reduces the experimental barrier for quantitatively characterizing electron-ion entanglement. This practical simplification can facilitate broader investigations into quantum entanglement, thereby promoting routine exploration of entanglement effects across various ultrafast experiments.

An essential insight gained from our energy-resolved purity analysis is the recognition of subtle spectral interferences emerging from multi-electron resonant states. Our findings reveal intricate substructures arising from coherent interactions between energy-dependent partial-wave channels. These interference patterns substantially alter the purity distribution, reflecting a rich interplay of constructive and destructive interference effects. Such spectral fine structures, which become apparent only under careful multi-electron treatment, open intriguing opportunities for exploring even finer-scale electron dynamics in future experiments.

The quantitative determination of scattering-phase differences among distinct multi-electron states represents another critical achievement of our study. Extracting these phases explicitly from delay-dependent PAD oscillations provides a direct connection between experimentally observable angular distributions and fundamental short-range electron-ion interactions. By directly probing these phases through our PAD-based methodology, future experimental work can systematically explore electron-ion interaction potentials, enriching our fundamental understanding of electron scattering processes in complex atomic and molecular environments.

While the present demonstration employs argon atoms as a prototypical system, the broader applicability of our method invites intriguing extensions. For instance, in linear molecular systems, the atomic $({s}_{0},{d}_{0},{d}_{\pm 1})$ basis maps naturally to $\sigma$ and $\pi$ continua. For general molecules, the channel labels become point-group irreducible representations of the ionic core coupled to continuum partial waves. As long as the intermediate state has energy-splitting due to orbital angular momentum coupling. The delay-independent-PAD workflow yields a quantitative readout of these effects in molecules. Moreover, the coupling of electronic and nuclear degrees of freedom can significantly modify electron-ion correlation dynamics, introducing phenomena such as symmetry-dependent ionization pathways or chirality-dependent entanglement signatures^46,47^. Extending our coherent manipulation framework to these more complex targets could substantially advance molecular attosecond physics, revealing new dimensions of quantum correlation effects unique to molecular environments. Specifically, investigating photoelectron circular dichroism^48,49^ and related chiral effects in molecular ionization scenarios could yield powerful new diagnostics of entanglement that are sensitive to both electronic structure and nuclear dynamics. From a broader perspective, our demonstrated capability for coherent control and precise measurement of electron-ion entanglement has profound implications for emerging quantum technologies and attosecond science. Integrating our entanglement-based control methods with advanced ultrafast and FEL facilities^35^ could significantly enhance experimental capabilities, facilitating deeper exploration of correlated electron dynamics in atoms, molecules, and condensed matter systems. By bridging theoretical sophistication with experimental practicality, our work sets the stage for transformative advancements in the characterization and application of quantum entanglement within the broader ultrafast science community.

## Materials and methods

### Multi-configurational time-dependent Hartree-Fock method

We apply the MCTDHF method^38–41^ to solve the multi-electron time-dependent Schrödinger equation (ME-TDSE) of an argon atom exposed to an external field within the dipole approximation

29 $$i\frac{d}{{dt}}\Psi \left(t\right)=H\left(t\right)\Psi \left(t\right)$$

where the Hamiltonian is

30 $$H\left(t\right)=\mathop{\sum }\limits_{j=1}^{{N}_{e}}h\left(j\right)+\frac{1}{2}\mathop{\sum }\limits_{j=1}^{{N}_{e}}\mathop{\sum }\limits_{k\ne j}^{{N}_{e}}U\left(j,k\right)$$

with ${N}_{e}$ denoting the number of electrons and

31 $$h\left(j\right)=-\frac{1}{2}{{\boldsymbol{\nabla }}}_{j}^{2}+{\boldsymbol{E}}\left(t\right)\cdot {{\boldsymbol{r}}}_{j}+V\left({r}_{j}\right)$$

32 $$U\left(j,k\right)=\frac{1}{\left|{{\boldsymbol{r}}}_{j}-{{\boldsymbol{r}}}_{k}\right|}$$

To reduce the computational cost, the Coulomb potential $V$ is implemented as an effective core potential (ECP) which describes the screening of the inner-shell electrons so that only the outermost eight electrons in $3s$ and $3p$ shells are involved in practical calculations. We choose M. Dolg’s ECP^50^, which reads $V(j)=\mathop{\sum }\limits_{l}{O}_{l}(j){V}_{l}({r}_{j}){O}_{l}(j)$, where

33 $${O}_{l}=\mathop{\sum }\limits_{m=-l}^{l}\left|{lm}\right\rangle \left\langle {lm}\right|$$

34 $${V}_{l}\left(r\right)=\left\{\begin{array}{lc}-\dfrac{{Z}_{{\rm{c}}}}{r}+\mathop{\sum }\limits_{j}{D}_{l,j}\exp \left(-{d}_{l,j}{r}^{2}\right) & l\le {L}_{{\rm{c}}}\\ {V}_{{L}_{c}}\left(r\right) & l > {L}_{{\rm{c}}}\end{array}\right.$$

${Z}_{{\rm{c}}}=8$ is the charge number of the compound system of core electrons and nuclei. Typically, ${L}_{{\rm{c}}}-1$ refers to the largest angular momentum used by the core orbitals. The values of ${D}_{l,j}$ and ${d}_{l,j}$ can be found in Ref. ^51^. We note that $V$ is non-local, thus not a function of ${\boldsymbol{r}}$. The vector potential ${\boldsymbol{A}}(t)$ satisfying ${\boldsymbol{E}}(t)=-\partial {\boldsymbol{A}}(t)/\partial t$ is

35 $${\boldsymbol{A}}\left(t\right)={A}_{1}g\left(\frac{t}{{T}_{1}}\right)\sin \left({\omega }_{1}t\right){{\boldsymbol{e}}}_{z}+{A}_{2}g\left(\frac{t-\tau }{{T}_{2}}\right)\sin \left({\omega }_{2}t\right){{\boldsymbol{e}}}_{z}$$

where ${A}_{q}$, ${T}_{q}$, and ${\omega }_{q}$ are the peak amplitudes, foot-to-foot widths, and angular frequencies of the first pulse ($q=1$) and the second pulse ($q=2$) in the two-stage scheme. $\tau$ is the delay between the two pulses and

36 $$g\left(\lambda \right)=\left\{\begin{array}{cc}{\sin }^{2}\left(\pi \lambda \right) & \lambda \in \left[0,1\right]\\ 0 & \lambda \,\notin \,\left[0,1\right]\end{array}\right.$$

Within the MCTDHF theory, the many-electron wave function is decomposed into a series of Slater determinants as

37 $$\left|\Psi \left({\rm{t}}\right)\right\rangle =\mathop{\sum }\limits_{I\in {\rm{CAS}}}{C}_{I}\left(t\right)\left|\varPhi \left(t\right)\right\rangle$$

Here, ${C}_{I}(t)$ are the expansion coefficients, and $|\varPhi \left(t\right)\rangle$ are the configuration state wave functions in the complete active space (CAS) spanned by a set of time-dependent single-particle orbitals $\{{\phi }_{j}(t)\}$. Moreover, only configuration states with $M=0$ are included since only linearly polarized fields are applied. The spin-restrictive formalism is used throughout. In this work, up to $13$ spatial orbitals are used, corresponding to a maximal configuration number of $78095$.

The equations of motion (EOMs) of MCTDHF derived from the time-dependent variational principle^52–54^ read

38 $$i\frac{d{C}_{I}\left(t\right)}{{dt}}=\mathop{\sum }\limits_{J\in CAS}\left\langle {\varPhi }_{I}\left|\hat{H}\right|{\varPhi }_{J}\right\rangle {C}_{J}(t)$$

39 $$i\frac{d}{{dt}}\left|{\phi }_{j}\right\rangle =\hat{Q}\hat{F}|{\phi }_{j}\rangle$$

where $\hat{Q}=1-{\sum }_{j}|{\phi }_{j}\rangle \langle {\phi }_{j}|$. A detailed definition of the generalized Fock operator $\hat{F}$ can be found in Ref. ^54^. The above EOMs are solved by an ETD-RK4^55^ solver after discretization. We expand the single-particle functions with a set of eighth-order B-spline functions as

40 $${\phi }_{j}\left({\bf{r}},t\right)=\mathop{\sum }\limits_{ln}{c}_{{jln}}\left(t\right)\frac{{B}_{n}\left(r\right)}{r}{Y}_{l{m}_{j}}\left({\theta }_{r},{\phi }_{r}\right)$$

where ${B}_{n}(r)$ and ${Y}_{{lm}}({\theta }_{r},{\phi }_{r})$ are the set of B-spline functions and spherical harmonics. Here, ${m}_{j}$ is given fixed for the $j$-th orbital. The calculations are conducted in a spherical box with a radius of $1600$ a.u., discretized by $3520$ B-spline functions for each spherical partial wave. The maximum orbital angular momentum is $12$. The time step size for the real-time propagation is $0.008$ a.u.

At the end of the propagation, the part of each single-particle orbital in the asymptotic region is projected onto plane waves for extracting the momentum information. The process is detailed in Ref.^56^. A weighted sum, based on the density matrix of the single-particle orbitals, of these projections can lead to the final PMDs. Similarly, information about partial waves can be obtained.

### R-matrix with time-dependence method

Cross-validation with the non-relativistic RMT method is performed in this study. A comprehensive description of the RMT methodology can be found in Ref. ^42^, while the treatment of target states (Ar^+^ in this work) is detailed in Ref. ^57^. The RMT simulations are conducted in a spherical box with a radius of $2832$ a.u. The inner region occupies a volume with a radius of $20$ a.u. The maximum orbital angular momentum is set to $8$, while the total orbital magnetic quantum number is constrained to $0$. The wave function is propagated with a time step size of $0.01$ a.u.

In RMT calculations, a manual energy offset is applied to align the ground state energy with experimental values. The energy differences between the ground state and the intermediate resonant ^1^D^e^ and ^1^S^e^ states are $12.90$ eV and $13.12$ eV, respectively, leaving a term splitting of $0.22$ eV (see supplementary information), which corresponds to a quantum beat period of $19$ fs. To prepare a near-equal superposition of the resonant states, the first pulse frequency is set to ${\omega }_{1}=6.52$ eV for two-photon excitation, with an intensity of $1\times {10}^{13}$ W/cm^2^ and a foot-to-foot duration of 32 cycles. Further details regarding the access to the resonant states and the rationale for the first pulse frequency can be found in the supplementary information.

The second pulse has a frequency of ${\omega }_{2}=9.52$ eV, with a foot-to-foot duration of $44$ cycles. The intensity matches the first pulse. Note that the frequency of the second pulse is flexible, as primarily required to ionize the system from the resonant states while avoiding overlap with the signals at $(2+n){\omega }_{1}$ purely induced by the first pulse. We vary $\tau$ from $20$ fs to $40$ fs, encompassing a full quantum beat period estimated before. Notably, when $\tau \ge 20$ fs, the pulses are temporally non-overlapping, effectively suppressing ionization via simultaneous absorption of two ${\omega }_{1}$ photons and one ${\omega }_{2}$ photon, which would otherwise produce $f$ waves of the photoelectron.

### SAE-TDSE method

Simulations under the SAE approximation are performed using the QPC-TDSE program^56^. The Tong-Lin potential is adopted to model the electrons in the $3$*p*-shell of argon atoms^58^. Details of the potential parameters are found in Ref. ^59^. The orbital energies for the $3p$ and $4p$ states are $-15.76$ eV and $-2.59$ eV, respectively. In this case, we set ${\omega }_{1}=6.585$ eV to excite the system to the $4p$ state. Other laser parameters are kept the same as those specified in the RMT calculations.

The simulation is performed inside a spherical box with a radius of $2500$ a.u., with each radial component expanded via $8000$ eighth-order B-spline functions. The maximum orbital angular momentum is set to $12$. The Crank-Nicolson propagator is employed with a time step size of $0.008$ a.u. The final states are projected onto exact scattering states of the effective potential to compute the PMDs immediately after the laser pulse ends. The contributions of photoelectron that departs from $3{p}_{0}$, $3{p}_{1}$ and $3{p}_{-1}$ orbitals are considered.

## Supplementary information

Supplementary Information

## Data Availability

The data that support the findings of this work are openly available at https://scidata.sjtu.edu.cn/records/ecepr-c4065.
