# Supplementary material for: Coherent control of electron-ion entanglement in multiphoton ionization
Source: Light Sci Appl. 2026 Mar 6;15:156. doi: 10.1038/s41377-025-02151-y (PMC12966377; doi:10.1038/s41377-025-02151-y)
Supplement: Supplementary file 1 — Supplementary Information [file 41377_2025_2151_MOESM1_ESM.pdf]

Supplementary Information for

Coherent control of electron-ion entanglement in multiphoton ionization

Yi-Jia Mao[1,2†], Zhao-Han Zhang[2†], Yang Li[2*], Takeshi Sato[3,4,5],

Kenichi L. Ishikawa[3,4,5,6], Feng He[1,2*]

[1]Tsung-Dao Lee Institute, Shanghai Jiao Tong University, Shanghai 201210, China.

[2]Key Laboratory for Laser Plasmas (Ministry of Education) and School of Physics and Astronomy, Collaborative Innovation Center for IFSA (CICIFSA), Shanghai Jiao Tong University, Shanghai 200240, China.

[3]Department of Nuclear Engineering and Management, Graduate School of Engineering, The University of Tokyo,7-3-1 Hongo, Bunkyo-ku, Tokyo 113-8656, Japan.

[4]Photon Science Center, Graduate School of Engineering, The University of Tokyo, 7-3-1 Hongo, Bunkyo-ku, Tokyo 113-8656, Japan.

[5]Research Institute for Photon Science and Laser Technology, The University of Tokyo, 7-3-1 Hongo, Bunkyo-ku, Tokyo 113-0033, Japan.

[6]Institute for Attosecond Laser Facility, The University of Tokyo, 7-3-1 Hongo, Bunkyo-ku, Tokyo 113-0033, Japan.

[*]Corresponding authors. Email: liyang22@sjtu.edu.cn; fhe@sjtu.edu.cn

[†]These authors contributed equally to this work.

1 Derivation of the photoelectron purity

When spin-orbit coupling is neglected, the spin polarizations of the photoelectron and the ion are conserved. We can then disregard the degree of spin in the following discussion. The total wave function of the combined ion and photoelectron system can be decomposed under the decoupled representation as

$$|\Psi\rangle = \sum_{m_i,m_e} C_{m_i,m_e} |m_i\rangle |m_e\rangle . \tag{S1}$$

C_{m_i,m_e} denotes the amplitude of each decoupled state, where the contributions from different l_e but the same m_e are already summed up. Although both the ionization from the $3s$ and $3p$ orbitals are considered, for the ionization signal near $\varepsilon = 2\omega_1 + \omega_2 - I_p$, the contributions primarily originate from the $3p$ shells. Therefore, the angular quantum number of the ion, l_i, can always be considered as 1, which can be omitted in the decomposition of the total wave function.

Under linearly polarized fields, the conservation of the total magnetic quantum number ($m_i + m_e = 0$) further simplifies the decomposition to

$$|\Psi\rangle = \sum_{m_i} C_{m_i} |m_i\rangle |-m_i\rangle . \tag{S2}$$

The density matrix of the system is calculated as

$$\begin{aligned} \rho &= |\Psi\rangle\langle\Psi| \\ &= \sum_{m_i,m_i'} C_{m_i} C_{m_i'}^{*} |m_i\rangle |-m_i\rangle \langle -m_i'|\langle m_i'|. \end{aligned} \tag{S3}$$

By taking the partial trace with respect to the ionic state, the density matrix of the photoelectron can be obtained as

$$\begin{aligned} \rho_e = \mathrm{Tr}_i[\rho] &= \sum_{m_i''} \sum_{m_i,m_i'} C_{m_i} C_{m_i'}^{*} \langle m_i'' |m_i\rangle |-m_i\rangle \langle -m_i'|\langle m_i'|m_i''\rangle \\ &= \sum_{m_i''} \sum_{m_i,m_i'} C_{m_i} C_{m_i'}^{*} \delta_{m_i'',m_i} \delta_{m_i',m_i''} |-m_i\rangle \langle -m_i'| \\ &= \sum_{m_i''} |C_{m_i''}|^2 |-m_i''\rangle \langle -m_i''| \\ &= \sum_{m_e} |C_{m_e}|^2 |m_e\rangle \langle m_e|. \end{aligned} \tag{S4}$$

It is clearly observed that the electronic subsystem is in mixed states. For the ionization of argon atoms induced by linearly polarized fields, $m_e = 0, \pm 1$. At the scattering energy range of interest, up to the d wave dominates the photoelectron energy spectrum. As mentioned before, the probabilities, $|C_{m_e}|^2$ contain the contributions with the same m_e but different angular quantum numbers. Therefore, the probabilities can be estimated by the partial-wave probabilities as

$$|C_0|^2 = \frac{M_{s_0} + M_{d_0}}{M_{s_0} + M_{d_0} + 2M_{d_1}}, \tag{S5}$$

$$|C_{+1}|^2 = |C_{-1}|^2 = \frac{M_{d_1}}{M_{s_0} + M_{d_0} + 2M_{d_1}}. \tag{S6}$$

These probabilities are normalized to ensure that $\mathrm{Tr}[\rho_e] = 1$. The factor of 2 in M_{d_1} accounts for the equivalent contributions for the d_1 and d_{-1} waves. Then, the purity of the scattering electron is

$$P_e = \mathrm{Tr}[\rho_e^2] = \frac{(M_{s_0} + M_{d_0})^2 + 2M_{d_1}^2}{(M_{s_0} + M_{d_0} + 2M_{d_1})^2}. \tag{S7}$$

2 Derivation of anisotropy parameters

In the main text, we have derived that, for the $(2+1')$ REMPI signal via the p resonant states in argon atoms, the anisotropy parameters of the PAD are

$$b_0 = \frac{1}{4\pi}(M_{s_0} + M_{d_0} + 2M_{d_1}), \tag{S8a}$$

$$b_2 = \frac{5}{14\pi}(M_{d_0} + M_{d_1}) + \frac{\sqrt{5}}{2\pi}\mathrm{Re}[C_{s_0}^* C_{d_0}], \tag{S8b}$$

$$b_4 = \frac{3}{14\pi}(3M_{d_0} - 4M_{d_1}). \tag{S8c}$$

To build a quantitative connection between the quantum beat between the resonant states and the anisotropy parameters, we first calculate the partial-wave probabilities with the amplitudes of different coupled states, C_{ψ_j} (See main text). In the calculation, the phases of the complex amplitudes of different multi-electron states need to be written explicitly to show a clear dependence on the pulse delay. According to the definition of the phases regarding different states $|\psi_i\rangle$ in the main

text, the partial-wave probabilities can be calculated as

$$M_{s_0}(\tau) = |C_{\psi_3}|^2 = (\alpha_3^{\mathrm{D}})^2 + (\alpha_3^{\mathrm{S}})^2 + 2\alpha_3^{\mathrm{D}}\alpha_3^{\mathrm{S}} \cos(\Delta E\tau + \chi_{\mathrm{DS}}), \tag{S9a}$$

$$\begin{aligned} M_{d_0}(\tau) &= \left| \sqrt{\frac{3}{5}} C_{\psi_1} - \sqrt{\frac{2}{5}} C_{\psi_2} \right|^2 \\ &= \left[\frac{3}{5}(\alpha_1^{\mathrm{D}})^2 + \frac{2}{5}(\alpha_2^{\mathrm{D}})^2 + \frac{2}{5}(\alpha_2^{\mathrm{S}})^2 - \frac{2\sqrt{6}}{5}\alpha_1^{\mathrm{D}}\alpha_2^{\mathrm{D}} \cos(\eta_{12}) \right] + \frac{4}{5}\alpha_2^{\mathrm{D}}\alpha_2^{\mathrm{S}} \cos(\Delta E\tau + \chi_{\mathrm{DS}}) \\ &\quad - \frac{2\sqrt{6}}{5}\alpha_1^{\mathrm{D}}\alpha_2^{\mathrm{S}} \cos(\Delta E\tau + \eta_{12} + \chi_{\mathrm{DS}}), \end{aligned} \tag{S9b}$$

$$\begin{aligned} M_{d_1}(\tau) &= \left| \sqrt{\frac{1}{5}} C_{\psi_1} + \sqrt{\frac{3}{10}} C_{\psi_2} \right|^2 \\ &= \left[\frac{1}{5}(\alpha_1^{\mathrm{D}})^2 + \frac{3}{10}(\alpha_2^{\mathrm{D}})^2 + \frac{3}{10}(\alpha_2^{\mathrm{S}})^2 + \frac{\sqrt{6}}{5}\alpha_1^{\mathrm{D}}\alpha_2^{\mathrm{D}} \cos(\eta_{12}) \right] + \frac{3}{5}\alpha_2^{\mathrm{D}}\alpha_2^{\mathrm{S}} \cos(\Delta E\tau + \chi_{\mathrm{DS}}) \\ &\quad + \frac{\sqrt{6}}{5}\alpha_1^{\mathrm{D}}\alpha_2^{\mathrm{S}} \cos(\Delta E\tau + \eta_{12} + \chi_{\mathrm{DS}}). \end{aligned} \tag{S9c}$$

Therefore, the anisotropy parameters can be calculated as

$$\begin{aligned} b_0(\tau) = \frac{1}{4\pi} \Big[&(\alpha_1^{\mathrm{D}})^2 + (\alpha_2^{\mathrm{D}})^2 + (\alpha_2^{\mathrm{S}})^2 + (\alpha_3^{\mathrm{D}})^2 + (\alpha_3^{\mathrm{S}})^2 \\ &+ 2(\alpha_2^{\mathrm{D}}\alpha_2^{\mathrm{S}} + \alpha_3^{\mathrm{D}}\alpha_3^{\mathrm{S}}) \cos(\Delta E\tau + \chi_{\mathrm{DS}}) \Big], \end{aligned} \tag{S10a}$$

$$\begin{aligned} b_2(\tau) = \frac{1}{\pi} \Big[&\frac{2}{7}(\alpha_1^{\mathrm{D}})^2 + \frac{1}{4}(\alpha_2^{\mathrm{D}})^2 + \frac{1}{4}(\alpha_2^{\mathrm{S}})^2 - \frac{\sqrt{6}}{14}\alpha_1^{\mathrm{D}}\alpha_2^{\mathrm{D}} \cos(\eta_{12}) - \frac{\sqrt{3}}{2}\alpha_1^{\mathrm{D}}\alpha_3^{\mathrm{D}} \cos(\eta_{13}) \\ &+ \frac{\sqrt{2}}{2}\alpha_2^{\mathrm{D}}\alpha_3^{\mathrm{D}} \cos(\eta_{23}) + \frac{\sqrt{2}}{2}\alpha_2^{\mathrm{S}}\alpha_3^{\mathrm{S}} \cos(\eta_{23}) \\ &- \frac{\sqrt{6}}{14}\alpha_1^{\mathrm{D}}\alpha_2^{\mathrm{S}} \cos(\Delta E\tau + \eta_{12} + \chi_{\mathrm{DS}}) + \frac{1}{2}\alpha_2^{\mathrm{D}}\alpha_2^{\mathrm{S}} \cos(\Delta E\tau + \chi_{\mathrm{DS}}) \\ &- \frac{\sqrt{3}}{2}\alpha_1^{\mathrm{D}}\alpha_3^{\mathrm{S}} \cos(\Delta E\tau + \chi_{\mathrm{DS}} + \eta_{13}) + \frac{\sqrt{2}}{2}\alpha_2^{\mathrm{S}}\alpha_3^{\mathrm{D}} \cos(\Delta E\tau + \chi_{\mathrm{DS}} - \eta_{23}) \\ &+ \frac{\sqrt{2}}{2}\alpha_2^{\mathrm{D}}\alpha_3^{\mathrm{S}} \cos(\Delta E\tau + \chi_{\mathrm{DS}} + \eta_{23}) \Big], \end{aligned} \tag{S10b}$$

$$b_4(\tau) = \frac{3}{14\pi} \left[(\alpha_1^{\mathrm{D}})^2 - 2\sqrt{6}\alpha_1^{\mathrm{D}}\alpha_2^{\mathrm{D}} \cos(\eta_{12}) - 2\sqrt{6}\alpha_1^{\mathrm{D}}\alpha_2^{\mathrm{S}} \cos(\Delta E\tau + \chi_{\mathrm{DS}} + \eta_{12}) \right]. \tag{S10c}$$

By fitting $b_0(\tau)$ and $b_4(\tau)$ with respect to τ, the phases χ_{DS} and $\chi_{\mathrm{DS}} + \eta_{12}$ can be extracted, respectively. In addition, it should be noted that the fitting of $M_{d_0} + 2M_{d_1} = \frac{1}{4\pi}[(\alpha_1^{\mathrm{D}})^2 + (\alpha_2^{\mathrm{D}})^2 + (\alpha_2^{\mathrm{S}})^2 + 2\alpha_2^{\mathrm{D}}\alpha_2^{\mathrm{S}} \cos(\Delta E\tau + \chi_{\mathrm{DS}})]$ can also directly obtain χ_{DS}. The fitting results of the anisotropy

Table S1: **The fitting results of the anisotropy parameters or the combinations of different partial waves.** The fittings are all conducted with the function $f(\tau) = G\cos(\Delta E\tau + \gamma) + H$. The coefficients obtained are also connected with the amplitudes or the phases in the equations above.

	G		γ	
Targets	values	correspondence	values	correspondence
$-14\pi b_4/3$	1.053	$2\sqrt{6}\alpha_1^{\mathrm{D}}\alpha_2^{\mathrm{S}}$	−2.145	$\eta_{12} + \chi_{\mathrm{DS}}$
$4M_{d_1} - 3M_{d_0}$	1.066	$2\sqrt{6}\alpha_1^{\mathrm{D}}\alpha_2^{\mathrm{S}}$	−2.115	$\eta_{12} + \chi_{\mathrm{DS}}$
$4\pi b_0$	0.141	$2(\alpha_2^{\mathrm{D}}\alpha_2^{\mathrm{S}} + \alpha_3^{\mathrm{D}}\alpha_3^{\mathrm{S}})$	−0.026	χ_{DS}
$2M_{d_1} + M_{d_0} + M_{s_0}$	0.141	$2(\alpha_2^{\mathrm{D}}\alpha_2^{\mathrm{S}} + \alpha_3^{\mathrm{D}}\alpha_3^{\mathrm{S}})$	−0.026	χ_{DS}
$2M_{d_1} + M_{d_0}$	0.075	$2\alpha_2^{\mathrm{D}}\alpha_2^{\mathrm{S}}$	0.010	χ_{DS}
M_{s_0}	0.066	$2\alpha_3^{\mathrm{D}}\alpha_3^{\mathrm{S}}$	−0.066	χ_{DS}

parameters and the partial-wave probabilities are listed in Table S1. By taking the ratio between $\alpha_1^{\mathrm{D}}\alpha_2^{\mathrm{S}}$ and $\alpha_2^{\mathrm{D}}\alpha_2^{\mathrm{S}}$, we can find that $\alpha_1^{\mathrm{D}}/\alpha_2^{\mathrm{D}} = 5.79$. This illustrates the complete dominance of the transition from $^1\mathrm{D}^{\mathrm{e}}$ to $^1\mathrm{F}^{\mathrm{o}}$ over $^1\mathrm{D}^{\mathrm{e}}$ to $^1\mathrm{P}^{\mathrm{o}}$, which is an extension of Fano's propensity from the single-electron state to the multi-electron state.

3 Choice of the first pulse

To determine the frequency of the first pulse, ω_1, pulses with frequencies ranging from 6.31 eV to 6.69 eV are applied to induce the three-photon ionization in argon atoms. This range covers the photon energies that can induce the two-photon transition to the resonant states $^1\mathrm{D}^{\mathrm{e}}$ and $^1\mathrm{S}^{\mathrm{e}}$. Fig. S1 shows the intensities of the photoelectron signal integrated over the ionization peak in the vicinity of $\varepsilon = 3\omega_1 - I_p$ with different applied laser frequencies. The contributions from different photoelectron partial waves or the multi-electron states are displayed, respectively, in (a) and (b).

The two peaks shown in the total signal, which are also the peaks of the states $|\psi_2\rangle$ and $|\psi_3\rangle$ in Fig. S1, correspond to the ionization pathways via the two resonant $4p$ states. We can speculate from the photoelectron energy spectrum (PES) that the energy difference between the two states is around 0.218 eV. The pump pulse in the main text is chosen as 6.52 eV for two reasons. Firstly, the

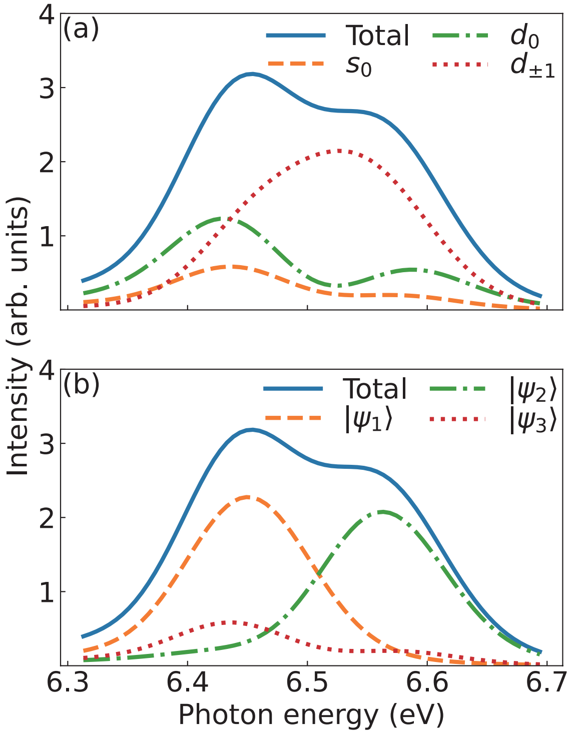

Fig. S1: **Three-photon ionization signal induced by the first pulse with different frequencies.** **a** Intensities of the photoelectron signal integrated over the ionization peak in the vicinity of $\varepsilon = 3\omega_1 - I_p$ with different applied pulse frequencies ω_1. The contributions for different partial waves are also provided. **b** Same as **a** but with contributions from different coupled channels.

contributions from $|\psi_1\rangle$ and $|\psi_2\rangle$ states are almost equivalent at this time, indicating a near-equal proportion of the ionization from the two resonant states. Due to the selection rule, $|\psi_1\rangle$ can only be transmitted from the $^1D^e$ state, while due to Fano's propensity rule, $|\psi_2\rangle$ is more likely to be ionized from the $^1S^e$ state. Secondly, at this frequency, the contribution of the d_0 wave is much lower than the $d_{\pm1}$ waves, which makes the change of the PADs in the sequential two-stage scheme more significant.

4 Comparison between RMT and MCTDHF results

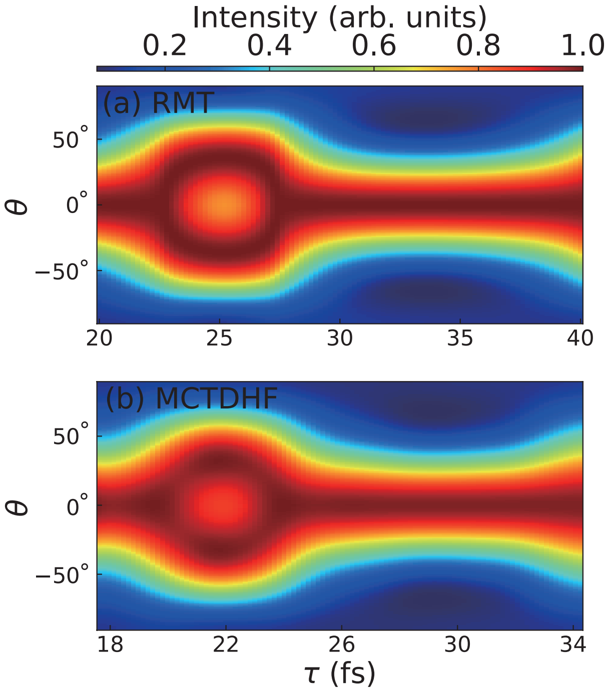

Fig. S2: **Comparison between the PADs calculated by the RMT method and the MCTDHF method. a** Delay-dependent PADs calculated by the RMT method. **b** Same as **a** but calculated by the MCTDHF method.

Figure S2 displays the comparison between the delay-dependent PADs calculated by the RMT method and those by the MCTDHF method. The behavior of the PADs as a function of the pulse from the two methods highly agrees with each other. A slight difference occurs in the oscillation periods and phases given by the two methods. Since the two methods apply different models, their estimations of the energies of the excited states and the scattering phases induced by the short-range

potential are different, which leads to the difference in the PADs.

5 Energy-resolved partial wave analysis

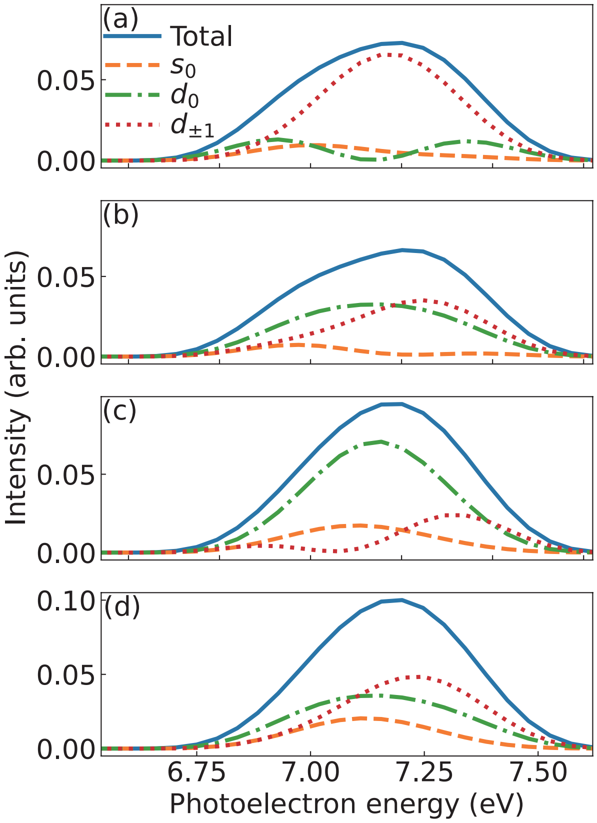

Fig. S3: **Photoelectron energy for different pulse delays. a** Photoelectron energy spectrum for $\tau = 24.75$ fs. The contributions from different partial waves are separated. **b, c, and d** Same as **a** but for $\tau = 29.5$, 34.25, and 39 fs.

In the PES of the $(2 + 1')$ REMPI, the peaks from different partial waves do not share the same center. Instead, their locations are sensitive to the pulse delay. Fig. S3 shows the ionization signal near $\varepsilon = 2\omega_1 + \omega_2 - I_p$. (a), (b), (c), and (d) show the total PES as well as the individual contributions from s_0, d_0, and $d_{\pm1}$ waves for pulse delays of 24.75, 29.5, 34.25, and 39 fs, respectively. It is discovered that $d_{\pm1}$ waves dominate the peak for $\tau = 24.75$ fs, whereas the situation reverses at the edges of the peak. This brings about three local maxima of purity in the energy domain. The case is similar for $\tau = 34.25$ fs, but the status of $d_{\pm1}$ and d_0 waves exchange with each other. Since $d_{\pm1}$

waves are actually two partial waves with equal contributions, when they dominate the process, the purity will be much smaller than the case where the d_0 wave dominates.

6 Extended behavior of the energy-resolved purity

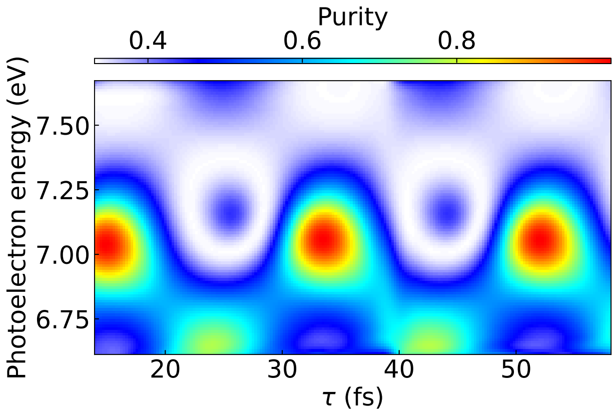

Fig. S4: **Energy-resolved purity as a function of the pulse delay with a larger delay range.** The delay range is extended to 14 – 58 fs.

In Fig. S4, we show the energy-resolved purity as a function of the pulse delay in the range of 14 – 58 fs. In this range, more than two oscillation periods are included. We can find that the purity also shows periodic behaviors as we expect due to the quantum beat between the two resonant states.
